# The Effect of Health and Economic Costs on Governments’ Policy Responses to COVID‐19 Crisis under Incomplete Information

**DOI:** 10.1111/puar.13394

**Published:** 2021-06-13

**Authors:** Germà Bel, Óscar Gasulla, Ferran A. Mazaira‐Font

**Affiliations:** ^1^ Universitat de Barcelona

## Abstract

The COVID‐19 pandemic has become an unprecedented health, economic, and social crisis. The present study has built a theoretical model and used it to develop an empirical strategy, analyzing the drivers of policy‐response agility during the outbreak. Our empirical results show that national policy responses were delayed, both by government expectations of the healthcare system capacity and by expectations that any hard measures used to manage the crisis would entail severe economic costs. With decision‐making based on incomplete information, the agility of national policy responses increased as knowledge increased and uncertainty decreased in relation to the epidemic's evolution and the policy responses of other countries.

**Evidence for Practice**

- Governments had incomplete information when they responded to COVID‐19.
- Confidence in healthcare‐system capacity and expected costs delayed their responses.
- Federal countries were more agile than unitary countries in developing policy responses.
- Healthcare‐system capacity does not fully guarantee epidemic management.

The coronavirus outbreak has produced an unprecedented health, economic, and social crisis, developing into a transboundary crisis, as characterized in the study by Boin (2019). Global leaders, including Antonio Guterres (Secretary General of United Nations) and Angela Merkel (Chancellor of Germany), have compared its impact to World War II.

In a crisis, authorities must engage in coherent analysis and search for proper responses, despite time limitations, uncertainty, and intense pressure (Boin et al. 2005); this has been the case during the COVID‐19 crisis (Van Dooren and Noordegraaf 2020). The rapid spread of the pandemic has forced countries to take unprecedented measures. More than 90 percent of the world's population lives in countries that have placed restrictions on people arriving from other countries. Many of these countries have closed their borders completely to noncitizens and nonresidents, according to the Pew Research Center (see Connor 2020). Quarantines, social distancing, and isolating infected populations can contain the epidemic.

There is no clear consensus on the specific impact of each measure used to mitigate propagation (see Anderson et al. 2020; Koo et al. 2020). At present, the literature includes few policy analyses related to COVID‐19. Among these, Moon (2020) has analyzed the policy response in Korea; Huang (2020) has shown that collaborative governance (cooperation between different levels of government and non‐governmental organizations) was a key factor in Taiwan's fight against COVID‐19; and Gupta et al. (2020) have analyzed behavioral responses to policies mandated in the United States. Any analysis of COVID‐19 policy is restricted, given the provisional character and limitations of the existing data (Stock 2020).

Despite this, there is a widespread consensus among researchers and international organizations that early prevention and response are critical (Grasselli, Pesenti, and Cecconi 2020), especially given the acute effect of pandemics on disadvantaged sectors of the population (Cénat et al. 2020; Deslatte, Hatch, and Stokan 2020; Furceri and Ostry 2020; Kapiriri and Ross 2020; Menifield, Charles, and Clark 2020; Scott, Crawford‐Browne, and Sanders 2016).

The available information allows us to analyze why some national policy responses have been more agile than others. Within the domain of policy decision‐making and implementation, agility is defined as “speed in responding to variety and change” (Gong and Janssen 2012, S61). Lai (2018, 459) defines agility as the “iterative, successive process of adjustment and routine‐breaking actions.” Agility is related to policy‐response quality (Lai 2018); it is also an aspect of robustness in policy design (Howlett, Capano, and Ramesh 2018). It has been a key factor in countries like South Korea, which has dealt with the COVID‐19 crisis successfully (Moon 2020). Agility is thus a relevant policy issue, as the time dimension is central to crisis management. The policies that governments have implemented to deal with COVID‐19 have followed distinct national (rather than consensual international) standards, in line with policy responses to previous epidemic crises (Baekkeskov 2016; Vallgårda 2007).

This article investigates why some countries took longer to institute lockdown measures than others. We present a model that characterizes the drivers of coronavirus reaction time, namely the number of known diagnosed cases per million people (incidence rate) when the government approved hard measures (partial or complete lockdowns). Our base model includes three main factors: the expected capacity of each health system to deal with the outbreak, the expected economic costs of hard measures, and the level of information available to governments forming these expectations. We extend our analysis to account for differences in governance and political regimes, emotional beliefs and biases affecting the assessment of pandemic‐related risk, and political survival factors.

We estimate an equation derived from our modeling. Using data from the OECD and European countries, we find that three main factors are statistically relevant. First, the government's expected capacity to fight the outbreak, measured as total healthcare expenditure per capita (adjusted for purchasing power parity), is a factor that delays policy response, accounting for 26.6 percent of the total delay. The higher a government's healthcare expenditure, the more it is likely to believe it can handle the outbreak—hence the longer delay in responding.

When it comes to preventing economic costs, the more a country is exposed to globalization and trade, the more (relatively) affected it will be by hard measures, such as border closures. We use total trade (% Gross Domestic Product) and the total travel and tourism contribution to GDP as proxies for the expected cost of hard measures. Both are highly significant; together, they account for 37.0 percent of the total predictive power of the model. As expected, the higher the cost, the slower the reaction.

To represent the level of information, we use the number of countries that instituted hard measures before a country experienced her first coronavirus cases. As expected, countries that experienced their first coronavirus cases when other countries already had lockdowns in place anticipated their responses. The level of information is responsible for 19.5 percent of the model's explanatory power. The evidence also confirms the relevance of decision‐making processes and types of decision makers. Concretely, federal states are more agile than unitary states.

In regard to emotional and perception‐related factors, proximity bias—represented by the distance from Wuhan to the capital city of each country—accounts for 5.9 percent of response agility. Finally, we extend our analysis by testing several variables related to values, ideological biases, and the political survival hypothesis, finding no systematic role for any of these factors.

The rest of the article is organized as follows. First, we outline the theoretical framework used to model the speed of response during the COVID‐19 outbreak and formulate empirical predictions, according to our model. Next, we discuss the data and present empirical results derived from our base equation. We extend the analysis by considering several additional hypotheses. We then conduct robustness checks. Finally, we draw our main conclusions and discuss some policy implications.

## Modeling the Decision of the Policy Response to the Crisis

We present a theoretical model developing an empirical strategy that we later follow to analyze the drivers of policy‐response agility. We begin with a basic model, representing a cost–benefit analysis carried out by a rational, benevolent government, which cares only about social welfare and has incomplete information on the pandemic. We then present two extensions. First, we allow for different types of decision makers (governments and political systems). Second, we consider the possibility that governments are (1) not entirely rational and potentially emotionally biased and (2) not fully benevolent, but driven by self‐interest (i.e., stay in office).

### Base Model: Benevolent Government with Incomplete Information

At the start of the pandemic, a set of natural features, such as the density of population (Wong and Li 2020), the share of population above 65 years old and with pre‐existing comorbidities (Álvarez‐Mon et al. 2021; Knight et al. 2020), temperature, and humidity (Mecenas et al. 2020), determine the virus reproductive number under no contention measures, ρ, and the death rate, *d*. The strategies used to fight the outbreak can be modeled as a sequential decision‐making process with incomplete information, where governments, instead of observing the true parameters involved in decision‐making, achieve only partial estimations. As noted in the introduction section, even after 7 months we lack clear knowledge of how the virus is propagated. We do not know how effective the various mitigation measures are (Stock 2020). Indeed, the very first response guidelines issued by the WHO in January 2020 were mainly addressed to communication and clinical management (WHO 2020a; WHO 2020b), and did not consider specific recommendations on contention measures, since due to the lack of information it was not even clear whether the virus was transmitted between humans.

In every time period, a government can decide to implement either hard or soft measures to contain the virus. If the government implements soft measures (SM) at time *t* (e.g., temperature control at airports or testing people with symptoms coming from affected countries), the transmission rate is reduced to *ρ*_*t*_ = *δ* ^*S*^ *ρ*. If it implements hard measures, it loses π units of utility (lost production) but reduces transmission rate to *δ* ^*H*^ *ρ*, with *δ* ^*H*^ < *δ* ^*S*^ < 1. It is worth highlighting that, according to cross‐country estimates (Hilton and Keeling 2020; Katul et al. 2020), all countries in our sample, no matter natural determinants, had reproductive numbers far above 1 (different methodologies lead to estimates ranging from 2 to 6.5), which imply that the pandemic would collapse their healthcare system unless massive tracking and severe contention measures were taken.^1^

Let *n*_*t* − 1_ be the number of infected people at the end of time *t* − 1. At the beginning of period *t*, the virus infects *ρ*_*t*_ *n*_*t* − 1_ people, who are then treated. Let the capacity of the healthcare system be c. If *n*_*t* − 1_ < *c*, then no infected people will die at *t* and all will be cured. Otherwise, the number of fatalities at *t* is *f*_*t*_ = *d*(*n*_*t*_ − *c*), and the rest are cured. The capacity of the healthcare system, while relevant in the direct sense of treating patients and avoiding fatalities, also influences the transmission rate by identifying and correctly diagnosing patients, thus breaking propagation chains. Hence, *ρ*_*t*_ must be seen as a function of contention measures, healthcare‐system capacity, and other potential country‐specific effects (e.g., hand‐washing habits).

**Figure 1:**
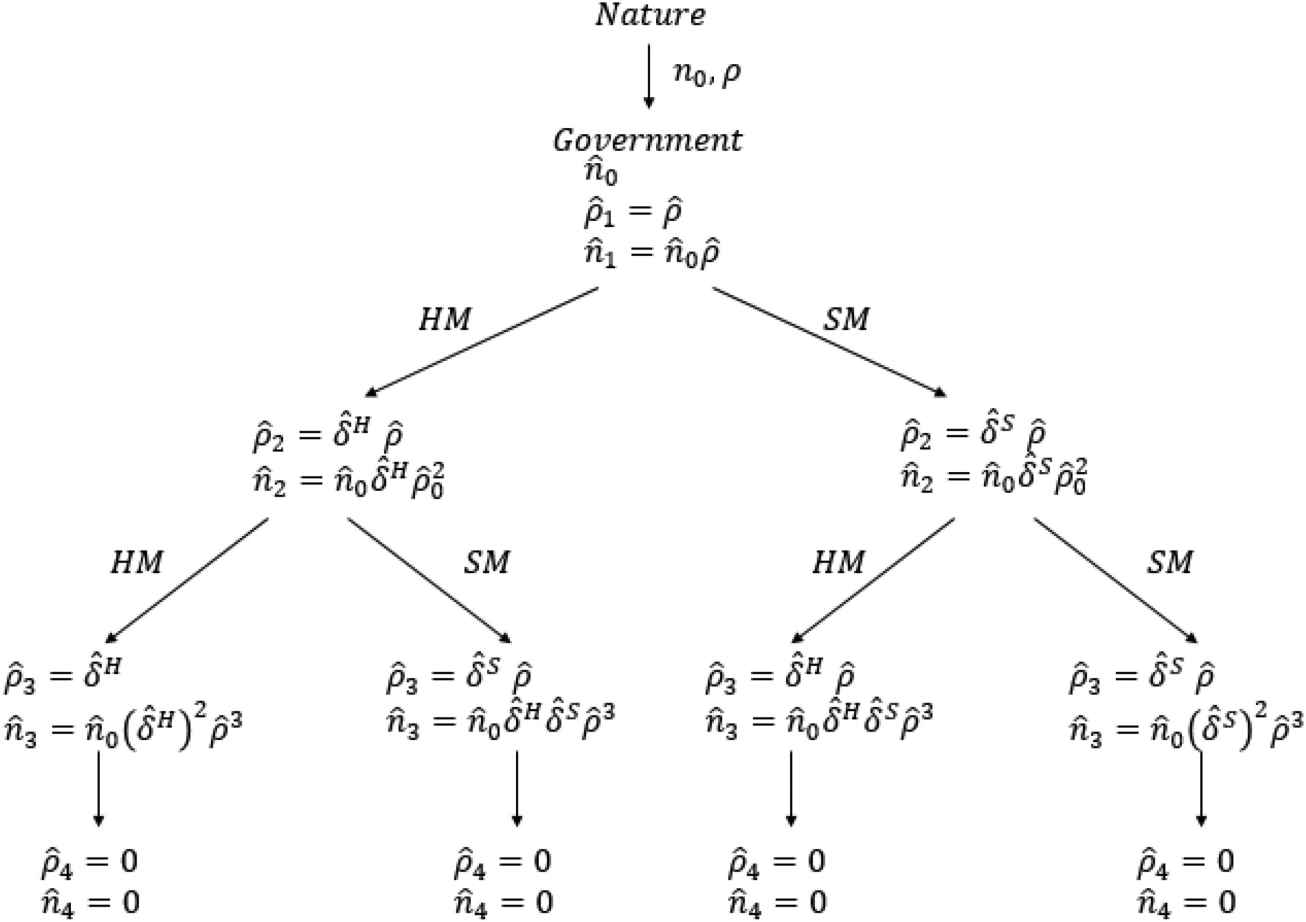
Representation of the 4‐Periods Decision Process with Government Expectation of Transmission Rates and Number of Infected People at *t* = 1

Let us consider a 4‐period process, as shown in Figure 1. At *t* = 0, nature determines an initial number of infected people *n*_*0*_ and the transmission rate *ρ*. At *t* = 1, infected people transmit the virus to others and then receive treatment. Therefore, *n*_1_ = *ρn*_0_, and the number of fatalities at *t* = 1 is *f*_1_ = *d* max{*n*_0_ − *c*, 0}. The government estimates the transmission rate $\rho_{1}=\hat{\rho }$ and the total number of infected people, $\hat{n_{1}}$. Based on that information, the government estimates the expected transmission rate, death rate, and healthcare‐system capacity during the following periods, as well as the impact and cost of various measures (${\hat{\rho }}_{t+1}=E_{t}\left(\rho_{t+1}\right)$, ${\hat{d}}_{t+1}=E_{t}\left(d_{t+1}\right)$, ${\hat{c}}_{t+1}=E_{t}\left(c_{t+1}\right)$, $\hat{\delta^{S}}=E_{t}(\delta^{S})$, $\hat{\delta^{H}}=E_{t}(\delta^{H})$, $\hat{\pi }=E_{t}\left(\pi \right)$). Based on these estimations, the government decides whether to implement soft or hard measures. The process continues until *t* = 4, when a vaccine is discovered and propagation drops to 0. Figure 1 shows how the government expects the pandemic to evolve, at *t* = 1.

Let us note, using ${\hat{f}}_{t+i}\left({\hat{n}}_{t}\right)={\hat{d}}_{t+i}\max\left\{{\hat{n}}_{t}-{\hat{c}}_{t+1},0\right\}$, the expected fatalities at time *t + i*, given the death‐rate and capacity expectations, and by *l* the cost per fatality. Given the information available to the government at *t* = 1, the expected costs (at *t* = 1) of various available strategies are as follows:

(1) $$EC\left(HM,HM\right)=l\left\{\begin{array}{l} {\hat{f}}_{1}\left({\hat{n}}_{{}_{0}}\right)+{\hat{f}}_{2}\left({\hat{\rho }}^{2}{\hat{n}}_{{}_{0}}\right)+{\hat{f}}_{3}\left({\hat{\rho }}^{2}\delta^{H}{\hat{n}}_{{}_{0}}\right)\\ +{\hat{f}}_{4}\left({\hat{\rho }}^{3}{\left(\delta^{H}\right)}^{2}{\hat{n}}_{{}_{0}}\right) \end{array}\right\}+2\pi$$

(2) $$EC\left(HM,SM\right)=l\left\{{\hat{f}}_{1}\left({\hat{n}}_{{}_{{}_{0}}}\right)+{\hat{f}}_{2}\left(\hat{\rho }{\hat{n}}_{{}_{{}_{0}}}\right)+{\hat{f}}_{3}\left({\hat{\rho }}^{2}\delta^{H}{\hat{n}}_{{}_{{}_{0}}}\right)+{\hat{f}}_{4}\left({\hat{\rho }}^{3}\delta^{H}\delta^{S}{\hat{n}}_{{}_{{}_{0}}}\right)\right\}+\pi$$

(3) $$EC\left(SM,HM\right)=l\left\{{\hat{f}}_{1}\left({\hat{n}}_{{}_{0}}\right)+{\hat{f}}_{2}\left(\hat{\rho }{\hat{n}}_{{}_{0}}\right)+{\hat{f}}_{3}\left({\hat{\rho }}^{2}\delta^{S}{\hat{n}}_{{}_{0}}\right)+{\hat{f}}_{4}\left({\hat{\rho }}^{3}\delta^{S}\delta^{M}{\hat{n}}_{{}_{0}}\right)\right\}+\pi$$

(4) $$EC\left(SM,SM\right)=l\left\{\begin{array}{l} {\hat{f}}_{1}\left({\hat{n}}_{{}_{0}}\right)+{\hat{f}}_{2}\left(\hat{\rho }{\hat{n}}_{{}_{0}}\right)+{\hat{f}}_{3}\left({\hat{\rho }}^{2}\delta^{S}{\hat{n}}_{{}_{0}}\right)\\ +{\hat{f}}_{4}\left({\hat{\rho }}^{3}{\left(\delta^{S}\right)}^{2}{\hat{n}}_{{}_{0}}\right) \end{array}\right\}$$

First, note that *EC(HM, SM) ≤ EC(SM, HM)*, with strict inequality, if the healthcare system collapses under soft measures. Delaying the adoption of hard measures is a weakly dominated strategy if the government expects a collapse. Therefore, under the assumption of rationality, a government will only delay implementation if it underestimates the risk or is overconfident about its healthcare‐system capacity, due to incomplete information. The latter reasoning is consistent with the offsetting behavior hypothesis, put forward by Peltzman (1975), which implies that risk is compensated for: agents adjust their behavior in response to perceived levels of risk and behave less carefully when they feel more protected. This hypothesis has been frequently tested, for instance, in car‐safety studies (Chirinko and Jr 1993; Peterson, Hoffer, and Millner 1995).

Hypothesis 1 The less information, the higher the probability of a delayed response.

Second, let us analyze what determines whether a government decides to implement hard or soft measures. The dynamics of government action or inaction during crises do not imply that action is always beneficial or functional (Rosenthal and Kouzmin 1997). Hence, governments must consider the costs and benefits of action (Comfort, Waugh, and Cigler 2012). A government will apply hard measures (at least once) if and only if the expected economic costs and fatalities are lower than they are predicted to be under soft measures (public debate on these interactions was well in the air already by mid‐March in the European countries most badly affected by the pandemics, Spain and Italy [Bel 2020; Ferro 2020).

It is sufficient to compare a case in which the government applies hard measures once. Noting via ∆*C* the difference between *EC(HM, SM)* and *EC(SM, SM)*, we have:

(5) $$\Delta C=l\left\{\begin{array}{l} {\hat{f}}_{3}\left({\hat{\rho }}^{2}\delta^{H}{\hat{n}}_{{}_{0}}\right)+{\hat{f}}_{4}\left({\hat{\rho }}^{3}\delta^{H}\delta^{S}{\hat{n}}_{{}_{0}}\right)-{\hat{f}}_{3}\left({\hat{\rho }}^{2}\delta^{S}{\hat{n}}_{{}_{0}}\right)\\ -{\hat{f}}_{4}\left({\hat{\rho }}^{3}{\left(\delta^{S}\right)}^{2}{\hat{n}}_{{}_{0}}\right) \end{array}\right\}+\pi$$

The more production a country expects to lose, because of hard measures, π, the fewer incentives the government has to implement hard measures, since ∆*C* increases as π increases. Note that fatality costs are positive only if the government believes that the system will collapse under soft measures. In that case, the incentives to implement hard measures increase.

Hypothesis 2 The greater the expected capacity of the healthcare system, the fewer incentives there are to implement hard measures.

Hypothesis 3 The higher the expected economic costs of hard measures, the fewer incentives there are to implement those hard measures.

Third, even if the system collapses, the government may decide not to implement hard measures. Let us assume that the system will collapse under soft measures at *t* = 3 and *t* = 4 and will never collapse under hard measures. Then:

(6) $$\Delta C=-l\left\{\;{\hat{f}}_{3}\left({\hat{\rho }}^{2}\delta^{S}{\hat{n}}_{0}\right)+{\hat{f}}_{4}\left({\hat{\rho }}^{3}{\left(\delta^{S}\right)}^{2}{\hat{n}}_{0}\right)\right\}+\pi$$

The government will implement hard measures if and only if the total number of fatalities multiplied by the cost per fatality is higher than the penalty cost of the hard measures. Therefore, the larger the process (all other things being equal), the higher the probability of hard measures.

Overall, this theoretical description of the decision‐making process, which assumes a welfare‐centered cost–benefit analysis, allows us to identify two main insights. First, the decision about which strategy to follow depends on the seriousness of the pandemic and the economic and fatality costs expected by the government. Governments may decide to follow different strategies because they expect different associated costs. Second, if a healthcare collapse is expected, it is better for a government to anticipate hard measures than to delay their implementation. In the present crisis, governments that instituted hard measures only after diagnosed coronavirus cases escalated would have been better off anticipating that policy response.

For the sake of simplicity, we assume that there is one type of hard measure. However, if a range of hard measures existed, the conclusions would be the same. The only difference would be that governments would choose a set of hard measures that minimized expected costs, according to the available information.

### Extension 1: Different Types of Decision Maker

So far, we have assumed that there is only one type of decision maker, the government, which operates with the same constraints and efficiency in every country. This is clearly not the case. Parliamentary systems and regimes have different decision‐making processes, both in terms of who makes decisions (the national government or both national and subnational governments) and how they are made and approved. For instance, a presidential regime is less dependent on approval from parliament to institute measures. For this reason, it may be able to react faster than a more institutionally complex governmental system. These differences may directly affect decision‐making agility and the implementation of measures. Debates have arisen over whether authoritarian governments have an advantage in responding to crises (Kleinfeld 2020; Schwartz 2012).

Let us use *g* to indicate the type of government and assume that there are two types: agile and slow. Agile governments (AG) resemble those modeled above: once they decide, the decision is approved and implemented during the next period. By contrast, slow governments (SG) face a more complex decision‐making and/or implementation process. They either need additional time to approve the measures (slow in decision‐making) or they fail to reduce the coronavirus transmission rate within a single time period, requiring two periods after the decision to apply hard measures (slow in implementation). Due to this delayed implementation—and coming back to the example in Figure 1—these governments slow down the transmission rate at *t* = 3 by applying hard measures at *t* = 1 and *t* = 2. For such governments, there is less incentive to implement hard measures. In conclusion, SG are expected to implement hard measures later than AG, when the decision‐process is more complex. They may also have fewer incentives to implement hard measures when their lack of implementation agility will reduce the expected benefit of such measures.

Hypothesis 4 The more presidential/executive a governance system is, the greater its ability to implement hard measures quickly.

### Extension 2: Emotions, Beliefs, and Political Survival

Alongside the uniformity of each country's decision‐making process and legal constraints, two other hypotheses can also be questioned. First, it may be wrong to assume that expectations will be rational; decision‐making can be highly influenced by emotions and beliefs, especially when information is lacking (Kahneman 2011). Akerloff and Shiller (2009) argue that emotions play a role in economics and are a key driver of market failures and financial crises. When decision makers confront a crisis with incomplete information, especially where policy responses involve unprecedented restrictions on human rights, emotional biases and beliefs related to risk‐aversion, information processing, and the role of government can affect response speed. For instance, the greater the geographic proximity of the crisis, the more it provides an incentive for policy action, based on heightened fear and attention (Nohrstedt and Weible 2010).

Hypothesis 5 Emotional biases and beliefs related to risk‐aversion, information processing, and the role of government affect response speed.

So far, we have assumed that policy makers only care about maximizing social‐welfare functions. However, an abundant literature shows that politicians behave as both citizens and candidates. In other words, while they do care about maximizing social welfare, they are also motivated by self‐interest, for example, winning or staying in office (Besley and Coate 1997; Osborne and Slivinski 1996). Applying the logic of political survival (de Mesquita et al. 2003) to crisis management, it follows that, since voters punish governments for improper crisis responses, risk‐averse governments will implement proactive policies, especially within highly competitive contexts and close to elections (Baekkeskov and Rubin 2014). In terms of modeling, we can modify the utility function to include a political reward *φ* > 0, which reduces the cost of hard measures, resulting in *π* − *φ*.

Hypothesis 6 Highly competitive contexts provide incentives for more agile policy responses.

## Variables, Data, and Sources

### Sample

To ensure a certain homogeneity between countries, our model considers the 36 OECD countries. We provide a robustness check by increasing the sample to include the five non‐OECD EU states (Bulgaria, Romania, Cyprus, Malta, and Hungary) and four EU‐candidate states (Albania, Montenegro, North Macedonia, and Serbia). Next, we discuss the variables used, based on the theoretical model. We explain how they are specified and what sources they are obtained from.

### Variables

#### Incidence Rate when Policy Response Began

We define the “incidence rate when the policy response began” as the number of coronavirus cases (according to the Johns Hopkins Coronavirus Resource Center) adjusted per total population at the point when the government began to implement hard measures. This variable captures the amount of time each government waited before implementing hard measures. Hard measures severely restrict the free movement of citizens (partial or total lockdowns). They include: closing borders; closing schools, universities, and public places; prohibiting public events and public gatherings; closing most or all nonessential shops; imposing curfews; and forcing people to work from home. It can be argued that these hard measures are of different intensity, either because of its nature or because they may not be applied nationwide.

To establish a more homogeneous criterion, at least such two measures must be in place for a country to be categorized as implementing hard measures. Table A1 presents the first hard actions taken by each country. The data were obtained from the IMF database of policy responses to COVID‐19 (https://www.imf.org/en/Topics/imf‐and‐COVID‐19/Policy‐Responses‐to‐COVID‐19) and the Think Global Health timeline (www.thinkglobalhealth.org/), in addition to official government websites and press briefings. Although a perfectly homogenous criterion may be not possible to establish, with the two measures threshold we ensure, for instance, that at the time of policy response, 60 percent of the countries had implemented a nationwide closure of nonessential shops, and 85 percent closed educational institutions.^2^

#### Fatality Costs and the Capacity of the Healthcare System to Fight the Outbreak

Total healthcare resources per capita (purchasing power parity—ppp) in 2017, the last available year, are used as a proxy for the government's expected healthcare‐system capacity, including fatality costs. Several statements made by political leaders have highlighted the relevance of healthcare‐system capacity in decision‐making—and expenditure as the primary proxy for healthcare‐system capacity. For instance, both Spanish Prime Minister Pedro Sánchez and French President Emmanuel Macron made public statements on (casually) the same day, March 10th, presenting their countries' robust healthcare systems as the best possible preparation for fighting the pandemic when they both were still sustaining that lockdown measures were not needed. Similarly, the Leader of the U.K. Labour Party, Keir Starmer, made a statement on May 7, establishing a direct causal link between the United Kingdom's higher incidence of coronavirus (in comparison to other European countries) and the Conservative government's cuts to healthcare expenditure. Data on healthcare expenditure per capita (ppp) have been obtained from the World Bank database (https://data.worldbank.org/indicator/SH.XPD.CHEX.PP.CD). While nominal expenditure can be strongly associated with different costs, adjusting for ppp allows makes it possible to control for cost differences. The results have been checked using alternative variables (healthcare expenditure as a % of GDP, a relative measure; and public healthcare expenditure as a % of GDP) to account for the direct capacity of public healthcare systems.

Although expenditure is a key indicator of a healthcare system's overall capacity and performance, and used as such by political leaders, this proxy may be, at least partially, inaccurate, as it does not reflect expenditure efficiency or reveal whether the expenditure has targeted areas relevant to fighting the pandemic. For this reason, we have carried out an additional robustness check by considering the Global Healthcare Security Index health indicator (https://www.ghsindex.org/) as an alternative measure of healthcare‐system capacity. Built by The Economist Intelligence Unit, the Johns Hopkins Center for Health Security, and the Nuclear Threat Initiative, the indicator measures a healthcare system's capacity to fight pandemic outbreaks, in terms of personnel deployment, hospital beds, capacity in clinics and community‐care centers, healthcare assessments, infection‐control practices, available equipment, and the ability to test and approve new medical countermeasures.

In accordance with Hypothesis 2, derived from the theoretical model and the offsetting behavior hypothesis (Peltzman 1975), we expect stronger healthcare‐system capacity to be negatively associated with policy‐response agility.

#### Economic Costs

When determining policies, governments consider their costs and benefits. The hard measures used to confront the COVID‐19 crisis, given their intrinsic characteristics, inevitably slow down business activity, damaging the economy. Trade and tourism are particularly damaged by measures that strongly restrict mobility. For instance, the Prime Travel Technology Index, which measures the performance of global‐technology companies in the travel and tourism industry, fell by more than 50 percent between mid‐February and mid‐March. By the end of July, prices were around 25 percent lower than in February (https://www.primeindexes.com/). By comparison, the MSCI World Index, which represents a broad cross‐section of global markets in all sectors, fell by 30 percent between mid‐February and mid‐March; by the end of July, prices were only 5 percent lower than in February (https://www.msci.com/). We therefore use two indicators to consider the relevance of economic costs: the total direct and indirect contribution of travel and tourism, and total trade (imports and exports), both as a percentage of total GDP in 2018. Both indicators have been obtained from the World Bank Database (https://data.worldbank.org/indicator/NE.TRD.GNFS.ZS, https://tcdata360.worldbank.org/indicators/tnt.tot.contrib.gdp).

We hypothesize (Hypothesis 3) that the higher the economic cost of adopting hard measures, the less agile the government adopting them will be.

#### Uncertainty and Information

We use the number of countries that had announced or were implementing hard measures when their governments first began dealing with the pandemic (the first case diagnosed within the country) as the main indicator for the level of government information. We also use two alternative specifications. First, we use the number of countries previously affected by the COVID‐19 pandemic. Second, we restrict previously affected countries to those that share borders with the country in question, or are connected to it by less than 250 km of sea, with the exception of Japan, Australia, South Korea, and New Zealand, which are considered neighbors, due to their historical ties and strong economic relationships.

Any time that elapses after a crisis erupts gives the government a chance to adjust its response and reduce the risk of problems, such as cognitive overload or panic (Moynihan 2008). For this reason, countries in which the first case occurred relatively late are expected to have had more accurate information and a greater understanding of the risks involved, allowing policy makers to reduce the gap between planning and practice (Comfort 2007). As the theoretical model (Hypothesis 1) predicts, we expect them to have acted relatively quickly, taking advantage of the extra information and clearer calls for urgent action before the crisis escalated (Farazmand 2007).

### Types of Decision Makers

As the theoretical model states, different types of decision makers implement different policy responses to the COVID‐19 outbreak. We operationalize these differences with the following three variables:

#### Political Regime

Scores ranging from −1 (Parliamentary system) to 1 (Presidential system) represent various types of government. Semi‐presidential countries, such as France and Lithuania, are ranked as 0. To be defined as “presidential,” systems must have an executive presidency that is separate from the legislature. Semi‐presidential countries have both an executive presidency and a separate head of government, who leads the remaining executive; this individual is appointed by the president and accountable to the legislature. Parliamentarian governments have no executive presidency or head of state. The head of government leads the executive and must maintain the confidence of the legislature to remain in power. Data have been obtained from the institutional web pages of each country.

#### Multilevel Governance

The dummy variable equals 1 when the country has a unitary system and 0 when the system is federal (source: https://www.britannica.com/topic/political‐system/Federal‐systems). We have no clear expectation for this variable. While more vertical and hierarchical systems may respond more quickly (Yan et al. 2020) and federal systems can be highly dysfunctional (see for the United States, Maxeiner 2019), as hypothesized in Hypothesis 4, decentralization may also lead to more agility and effectiveness (Christensen, Lægreid, and Rykkja 2016). Multilevel systems with collaborative governance between different levels of government and non‐state institutions (Downey and Myers 2020; Huang 2020; Scavo et al. 2008; Schwartz and Yen 2017) provide incentives for more agile and effective responses, as noted in Hypothesis 6.

#### Authoritarianism

This study rates the level of authoritarianism in each country on a scale of 0–100, based on the Political Rights and Civil Liberties Index from Freedom House (https://freedomhouse.org/countries/freedom‐world/scores); the scale ranges from 0 (no political rights and civil liberties) to 100 (full political rights and civil liberties). Thus, a country with a score of 80 in the Freedom House Index receives a score of 20 for authoritarianism. We do not have a clear expectation for this variable, as in the former case.

#### Tenure of the Prime Minister

We use the number of days since the PM took office as a proxy for her experience and decision‐making determination. Data have been obtained from the institutional web pages of each country. Experienced decision makers are expected to be more agile, as they are more aware of electoral punishment (Bechtel and Hainmueller 2011).

#### Coalition Government

The dummy variable equals 1 when a country's national government is formed by two or more parties, and 0 otherwise (source: institutional webpages). We expect collation‐based governments to be less agile, given the transaction costs of crossed monitoring and control between different parties in government (Thies 2001).

### Emotions, Beliefs, and Political Survival

We consider several variables related to emotional biases, beliefs, and the logic of political survival, following the discussion in the theoretical section above.

#### Proximity Bias on Information Processing

We consider the distance in *kilometers from Wuhan, China* to the capital city of each country (source Google Maps API), as a proxy for geographic‐proximity bias. For decision makers affected by emotional biases (Hypothesis 5), we expect countries closer to Wuhan to demonstrate more agile policy responses. The variable is included as the logarithm of the distance needed to capture a concave dissipation effect.

#### Gender Bias on Risk Aversion

The second indicator corresponds to the gender of the Prime Minister. The question of whether female prime ministers have taken faster and more executive action has been widely discussed (e.g., *CNN*, April 16, 2020; *The Guardian*, 25 April 2020). One possible explanation is that women are more risk‐averse than men and value safety more highly, as Barnes and Beaulieu's (2019) survey experiment on women and risk aversion argues. We specify the variable *Gender PM* as a dummy that takes value 1 for women and 0 otherwise (source: countries' official web pages). We expect female prime ministers to demonstrate more agile policy responses.

#### Ideology

To account for the possibility that different ideologies or beliefs about the role of government can influence how crises are viewed and managed (Dror 1994), we consider the ideology of the main political party in the national government, as this party has the primary role in the decision‐making process, even (to some extent) in federal countries. A scale ranging from −1 (left) to 1 (right) is used to represent the ideological position of the Prime Minister's party. Center parties are ranked as 0 (main sources: the World Bank Database of Political Institutions https://datacatalog.worldbank.org/dataset/wps2283‐database‐political‐institutions, and international alliances that include governing parties). Where ideological beliefs play a role (Hypothesis 5), we expect left‐wing parties to demonstrate more agile policy responses, as they tend to be more concerned with inequality. Pandemics attack the most disadvantaged segments of society with particular intensity (Deslatte, Hatch, and Stokan 2020; Kapiriri and Ross 2020).

#### Days to Next Election

Applying the logic of political survival (de Mesquita et al. 2003) to disaster management suggests the following: since voters punish governments for improper crisis responses, risk‐averse governments will implement proactive policies, especially within highly competitive contexts and close to elections (Baekkeskov and Rubin 2014). Among the hypotheses presented here, one has particular interest for our research: the relationship between policy responses and the electoral cycle. As our theoretical model (Hypothesis 6) suggests, the closer a government is to its next election, the more comprehensive its policy response will be (Bechtel and Hainmueller 2011). The variable “days to next election” corresponds to the logarithm of the number of days between the first diagnosed case of coronavirus in the country and the next scheduled or expected nationwide election date (sources: National Democracy Institute database https://www.ndi.org/ and countries’ official websites).

Table 1 describes the variables and their sources. Table 2 presents descriptive statistics. Table A2, in Appendix, presents the correlation matrix.

**Table 1:** Variables: Description and Sources

	Description	Source
*Dependent variable*		
Incidence rate	The number of diagnosed cases adjusted per million inhabitants when the government began implementing hard measures.	IMF and Think Global Health
*Covariates*		
Health expenditure per capita (ppp) (LN)	Logarithm of total healthcare expenditure per capita in 2017 (ppp).	World Bank
Tourism	Logarithm of total travel and tourism contribution to GDP.	World Bank
Trade	Logarithm total trade ‐imports and exports‐ as % GDP.	World Bank
Previously locked countries	Total # of countries that had begun to implement hard measures when pandemic hits the country.	Own elaboration
Political regime	Score representing from −1 (Parliamentary system) to 1 (Presidential system)	Institutional webs
Unitary	Dummy variable that equals 1 if the state is Unitary and 0 if it is Federal	Encyclopedia Britannica
Authoritarianism	Score from 0 (full political rights and civil liberties) to 100 (no political rights and civil liberties) on level of authoritarianism of country	Freedom House Index
Tenure of the Prime Minister (LN)	Logarithm of # of days since the PM took office	Institutional webs
Coalitional government	Dummy variable that equals 1 if the national government is a coalition	Institutional webs
Km from Wuhan	Logarithm of the distance in kilometers between Wuhan and the capital city of the country	Google maps API
Gender of the Prime Minister	Dummy variable that equals 1 if the Prime Minister is a female	Institutional webs
Ideology	Score from −1 (left) to 1 (right) of the political orientation of the political party of the PM. Center parties are given a 0. The classification is based on international political alliances.	World Bank Database of Political Institutions and institutional webs
Days to next election	Logarithm of the number of days between the first diagnosed case in the country and the next scheduled or expected relevant election date	National Democracy Institute and institutional webs
*Alternative covariates*		
Health expenditure % GDP	Logarithm of the health expenditure as % GDP	World Bank
Public health expenditure % GDP	Logarithm of public health expenditure as % GDP	World Bank
GHS Health capacity	Health capacity score (0–100) to fight pandemic outbreaks	GHS Index
Previously affected countries	Total number of countries that had diagnosed cases when the pandemic hits the country	Own elaboration
Previously affected neighbors	Total number of neighboring countries that had diagnosed cases when pandemic hits the country	Own elaboration

**Table 2:** Descriptive Statistics

	Min	Max	Mean	St Dev
Incidence rate when policy response began	0.01	379.90	68.13	89.53
Health expenditure per capita (ppp)	7.03	9.28	8.24	0.53
Tourism (LN)	1.46	3.54	2.23	0.48
Trade (LN)	3.31	5.96	4.52	0.54
Previously locked countries	0	8.00	1.03	1.40
Political regime	−1	1	−0.61	0.73
Unitary state	0	1	0.78	0.42
Authoritarianism	0	68.00	10.25	12.87
Tenure of the Prime Minister (LN)	3.91	8.55	6.75	1.12
Coalitional government	0	1	0.50	0.51
Km from Wuhan (LN)	6.92	9.47	8.90	0.53
Gender of the Prime Minister	0	1	0.19	0.40
Ideology	−1	1	0.11	0.92
Days to next election (LN)	4.45	7.51	6.62	0.80
Health expenditure % GDP (LN)	1.44	2.84	2.14	0.27
Public health expenditure % GDP (LN)	1.04	2.22	1.77	0.32
GHS Health capacity (LN)	3.45	4.30	3.89	0.23
Previously affected countries	1	45.00	21.22	12.41
Previously affected neighbors	0	5.00	1.78	1.49

## Empirical Model and Results

Our empirical analysis is based on the theoretical model presented. First, we estimate the base model: a benevolent government. We then test potential extensions of the model, estimate a final model, carry out robustness checks, and interpret the results.

### Base Model

Agility in taking action (cases adjusted by total population when hard measures are taken) is affected by the healthcare system's ability to avoid fatalities and reduce the transmission rate, the cost of hard measures, and information accessible to the government on expected coronavirus deaths and transmission rates. As the previous section explains, the following variables are used to capture these drivers: healthcare expenditure per capita, tourism, trade, and previously locked‐down countries. We thus estimate a base model in the form:

(7) Cases=f(population,healthcare,tourism,trade,locked−downcountries)

A discrete modeling approach is appropriate, given the nonnegative discrete nature of the problem. A GLM with negative binomial distribution is used in this empirical approach. The negative binomial allows us to capture over‐ and under‐dispersion, providing more robust estimates of the parameters and standard errors than a Poisson distribution. We also use OLS to adjust an alternative specification of the model. To do this, we transform the target into the logarithm of the incidence rate. Although, for a general discrete problem, this approach may lead to non‐normality of residuals and fail to solve the relationship between variance and mean associated with counting problems (Lindsey 2000; Long 1997), in this case, once the transformation residuals can be considered normal (the *p* value is .2020 for the Shapiro–Wilk test and 0.1364 for the Anderson–Darling test) and homoscedastic (the White test for heteroscedasticity yields *p* value = .3346), the average variance‐inflation factor (VIF) is 1.34 and no individual VIF is above 2.

Table 3 presents the results using both modeling techniques. The two methods yield similar estimations of the parameters. In both cases, the theoretical hypotheses cannot be rejected for all parameters. Confidence that existing healthcare‐system capacity can deal with the crisis is associated with a higher incidence rate and thus negatively associated with policy‐response agility. In this regard, our result is consistent with the offsetting‐behavior hypothesis. Expectations of economic impact, if hard measures are delayed, are also negatively related to policy‐response agility. By contrast, increased information and reduced uncertainty are associated with more agile policy responses, as long as more countries have adopted hard measures.

**Table 3:** Estimated Parameters of the Models

	Negative Binomial (1)	OLS Robust (2)
Constant	−35.7629*** (3.2399)	−24.6835*** (3.9229)
*Healthcare capacity*	1.8814*** (0.3199)	1.9741*** (0.3779)
*Tourism*	1.7654*** (0.3086)	2.0864*** (0.3806)
*Trade*	1.4632*** (0.2760)	1.6666*** (0.3972)
*Locked countries*	−0.6307*** (0.1359)	−0.6597*** (0.2448)
N. Observations	36	36
R‐Squared		0.8167
F‐Test		5.174e‐11***
Residual/null deviance	0.6833	

Notes: Standard errors in brackets. The estimations are robust to the exclusion of Sweden, which followed a recommendation‐based approach, rather than a lockdown strategy. They are also robust to the exclusion of the United States, which can be considered an outlier, given its system of multilevel governance and high expenditure on healthcare. The estimated value of the coefficients, when these countries are excluded, varies less than 10 percent, relative to the estimation in Table 3. The significance levels remain the same.

* *p* < .1,

** *p* < .05,

*** *p* < .01.

The negative binomial distribution avoids transforming the target and guarantees a proper fitting for the counting outcome, without the assumption of residual normality. We therefore take it as our base model. Next, we check the results (Table 4) using alternative specifications for healthcare‐system capacity and level of information.

**Table 4:** Estimated Parameters of the Models with Alternative Specifications

	Base Model (1)	(3)	(4)	(5)	(6)	(7)
Constant	−35.7629*** (3.2399)	−28.3715*** (3.2480)	−23.5908*** (2.4836)	−26.5582*** (4.6241)	−38.9770*** (3.3164)	−41.9789*** (3.0826)
*Healthcare capacity*	1.8814*** (0.3199)				2.1605*** (0.3694)	2.4985*** (0.2795)
*Tourism*	1.7654*** (0.3086)	1.4700*** (0.3563)	1.4176*** (0.3682)	1.5804*** (0.3883)	1.9634*** (0.3473)	1.9613*** (0.3241)
*Trade*	1.4632*** (0.2760)	2.1681*** (0.3646)	1.9171*** (0.3430)	1.8864*** (0.3725)	1.6058*** (0.3507)	1.5988*** (0.3273)
*Locked countries*	−.6307*** (0.1359)	−.8000*** (0.1776)	−.9081*** (0.1657)	−1.0642*** (0.1568)		
*% GDP health*		2.7408*** (0.8381)				
*% GDP public health*			1.4059** (0.6372)			
*GHS index*				1.3924* (0.8457)		
*Affected countries*					−.0345* (0.0197)	
*Affected neighbors*						−.2883** (0.1135)
N. Observations	36	36	36	36	36	36
Residual/null deviance	0.6833	0.5652	0.5311	0.5046	0.6179	0.6406

Notes: Standard errors in brackets. In addition to testing alternative specifications of the main drivers in the base model, we also tested the relevance of additional second‐order effects related with the distribution of the costs, in accordance with reviewer suggestions. We tested whether the percentage contribution of Micro, Small and Medium Enterprises (MSME) to the economy (% of employment generated by MSMEs) or the percentage unemployment were relevant as a second‐order economic factor, and whether the percentage of the population over 65 was relevant as a second‐order fatality‐cost factor. These variables were not relevant. Including them in the model did not change the significance or order of magnitude of the other estimates. Data on the MSME contribution to employment were taken from Eurostat (https://ec.europa.eu/eurostat/statistics‐explained/pdfscache/45509.pdf) and institutional web pages for Australia, Canada, Mexico, and South Korea. No data were available for New Zealand, Israel, or Chile, due to differences in classification criteria. Data on the percentage of unemployment were obtained from the World Bank (https://data.worldbank.org/indicator/SL.UEM.TOTL.ZS). Data on the percentage of the population over 65 were obtained from the World Bank (https://data.worldbank.org/indicator/SP.POP.65UP.TO.ZS). Results available in Table A3, in Appendix.

* *p* < .1,

** *p* < .05,

*** *p* < .01.

Estimations using alternative specifications for healthcare‐system capacity and level of information yield results that are almost identical to those obtained with the base model—Estimation (1). The same thing happens when we run OLS Robust estimations (results available on request). When healthcare‐system capacity is measured in relative terms (Estimations 3 and 4), goodness of fit is slightly lower, revealing that the absolute level of healthcare resources (adjusted by ppp) is more relevant than the relative level. When the level of information is measured in previously affected countries (Estimations 6 and 7), the level of significance changes from *p* < .01 to *p* < .10 and *p* < .05. This shows that governments obtain more information from the strategies adopted by other governments than from any other source. It is worth noting that *Affected neighbors* provide more explanatory power than *Affected countries*, revealing a proximity effect, which will be discussed later.

### Extension 1: Types of Decision Maker

Starting from our base model, estimated using the negative binomial distribution (which avoids transforming the target and guarantees a proper fitting for a counting outcome without assuming the normality of residuals), we test the relevance of variables affecting the type of decision maker. The results are shown in Table 5. While unitary states are less agile than federal states, there is no sound evidence that presidential systems react faster. In addition, there is no evidence that other factors, coalitions, tenure of the PM, or authoritarianism influence government‐response agility. Notice that, although we find no evidence that authoritarianism influences agility, it may influence policy‐response severity. Indeed, Sweden, the only country able to sustain a recommendation‐based strategy, has the lowest authoritarianism score (0 out of 100).

**Table 5:** Estimations of Extensions of the Model with Types of Decision Maker

	Base Model (1)	(8)	(9)	(10)	(11)	(12)
Constant	−35.7629*** (3.2399)	−33.6977 *** (3.5063)	−37.3742 *** (3.0024)	−33.2863*** (4.0559)	−36.8153 *** (3.3536)	−35.4222 *** (3.2469)
*Healthcare capacity*	1.8814*** (0.3199)	1.7326*** (0.3261)	1.9742*** (0.2973)	1.6942*** (0.3733)	1.8645*** (0.3164)	1.8936*** (0.3189)
*Tourism*	1.7654*** (0.3086)	1.6977*** (0.3160)	1.9011*** (0.2792)	1.6793*** (0.3149)	1.8550*** (0.3108)	1.6932*** (0.3276)
*Trade*	1.4632*** (0.2760)	1.2396*** (0.3068)	1.4032*** (0.2530)	1.3224*** (0.3057)	1.4956*** (0.2737)	1.3679*** (0.3064)
*Locked countries*	−0.6307*** (0.1359)	−0.5632*** (0.1352)	−0.6677*** (0.1284)	−0.5699*** (0.1683)	−0.6579*** (0.1383)	−0.5933*** (0.1376)
*Political regime*		−0.3818 (0.2319)				
*Unitary state*			1.0113*** (0.3135)			
*Authoritarianism*				−0.0172 (0.0208)		
*PM Tenure*					0.1281 (0.1257)	
*Coalition*						0.2170 (0.3101)
N. Observations	36	36	36	36	36	36
Residual/null deviance	0.6833	0.7032	0.7443	0.6881	0.6929	0.6873

Notes: Standard errors in brackets. Following a referee suggestion we tested also whether the size of the country, measured as the total population (LN), was relevant. The parameter was found not significant. Data were obtained from the World Bank (https://data.worldbank.org/indicator/SP.POP.TOTL). Results available upon request.

* *p* < .1,

** *p* < .05,

*** *p* < .01.

### Extension 2: Emotions, Beliefs, and Political Survival

Finally, we test several hypotheses involving emotions, beliefs, and political survival, as discussed in the previous section. We test these hypotheses from our base model extended via the type of player. We consider the *unitary* variable only, since the parliamentary system is not relevant when included together with the *unitary* dummy. The results are presented in Table 6.

**Table 6:** Estimations of Extensions of the Model with Emotions, Beliefs and Political Survival

	Model Base (1) Extended with Type of Decision Maker (9)	(13)	(14)	(15)	(16)
Constant	−37.3742*** (3.0024)	−44.4938*** (3.1912)	−37.8580*** (3.3412)	−37.5440*** (2.9558)	−41.7941*** (2.6196)
*Healthcare capacity*	1.9742*** (0.2973)	2.0338*** (0.2712)	2.0301*** (0.3323)	1.9462*** (0.2937)	2.0790*** (0.2507)
*Tourism*	1.9011*** (0.2792)	1.9030*** (0.2568)	1.9299*** (0.2979)	2.0037*** (0.2789)	1.8751 *** (0.2386)
*Trade*	1.4032*** (0.2530)	1.3059*** (0.2377)	1.3981*** (0.2526)	1.4463*** (0.2502)	1.2296*** (0.2180)
*Locked countries*	−0.6677*** (0.1284)	−0.6670*** (0.1216)	−0.6568*** (0.1288)	−0.7285*** (0.1371)	−0.6133*** (0.1041)
*Unitary state*	1.0113*** (0.3135)	1.2142*** (0.3004)	1.0036*** (0.3139)	1.0219*** (0.3129)	1.2222*** (0.2717)
*Km form Wuhan*		0.7701*** (0.2308)			
*Gender PM*			−0.1167 (0.3586)		
*Ideology*				0.1741 (0.1420)	
*Days to next election*					0.6194*** (0.1402)
N. Observations	36	36	36	36	36
Residual/null deviance	0.7443	0.7923	0.7450	0.7539	0.8104

Notes: Standard errors in brackets. Although “trust in government” reflects public perception, rather than government's beliefs, it might also inform governments' beliefs on the potential acceptance of hard measures by population (Robinson et al. 2020). We investigated its relevance, using a ranking provided by the World Bank database, *Public Trust in Politicians* (https://govdata360.worldbank.org/). The variable is not relevant to response agility (results available in Table A4, in Appendix). This is consistent with findings in Mizrahi, Vigoda‐Gadot, and Cohen (2021) that during crises citizens value more transparency and responsiveness than trust. Like authoritarianism, however, trust may be relevant to response severity and a topic for further research. For example, Sweden was the only country able to sustain a recommendation‐based strategy; it may be significant that Sweden has one of the highest scores for “trust in government” (5.24 over 7 vs. an average of 3.59 for other countries) and the lowest score for authoritarianism (0 out of 100).

* *p* < .1,

** *p* < .05,

*** *p* < .01.

It is clear that the distance from Wuhan is a significant factor in determining policy‐response agility (Estimation 13). The further a country is from Wuhan, the slower its reaction, consistent with the geographic‐proximity hypothesis. By contrast, the variable for prime minister gender (Estimation 14) does not significantly affect policy‐response agility. This result is consistent with Pondorfer, Barsbai, and Schmidt (2017), who found no actual gender differences in risk preferences, but rather a perception based on stereotypes.

Estimation (15) shows that *ideology* has no significant influence on policy‐response agility in relation to the COVID‐19 crisis. Alongside the main decision maker's ideology, which can be thought of as a conjunctural belief, we have tested the relevance of more structural beliefs about the role of the state in relation to egalitarianism. We have used the World Bank GINI index (https://data.worldbank.org/indicator/SI.POV.GINI) and the population head count ratio at national poverty lines (https://data.worldbank.org/indicator/SI.POV.NAHC?locations=JP and https://data.oecd.org/inequality/poverty‐rate.htm) to operationalize this test. No significant evidence has been found (results are available in Table A4, in Appendix).

Finally, our findings on political survival (Estimation 16) are consistent with the hypothesis that the closer a government is to the next election, the more agile its policy response will be.

Note that in Estimations 8–16, all variables in the base model keep the same sign and level of significance. We can therefore conclude that the basic results are very stable throughout all estimations conducted in this section.

## Robustness Check and Final Model Interpretation

We conduct two robustness checks and estimate the final model. First, we check whether the base model and significant extensions are robust to the inclusion of new countries. We introduce to the sample the five non‐OECD EU states (Bulgaria, Romania, Cyprus, Malta, and Hungary) and four EU‐candidate states (Albania, Montenegro, North Macedonia, and Serbia).

As Table 7 shows, the base model is robust to the inclusion of additional countries (Estimation 17). Both the type of player extension and proximity bias are also robust (Estimation 19). However, the policy‐survival factor is not significant when additional countries are included.

**Table 7:** Robustness Check Including Additional Countries in the Sample

	Base Model OECD (1)	Base Model (17)	Extended Model OECD (18)	Extended Model (19)
Constant	−35.7629*** (3.2399)	−33.8892*** (2.6753)	−44.2591*** (2.8718)	−41.4189*** (3.2929)
*Healthcare capacity*	1.8814*** (0.3199)	1.8423*** (0.2713)	2.0619*** (0.2438)	1.9154*** (0.2591)
*Tourism*	1.7654*** (0.3086)	1.3735*** (0.2676)	1.8647*** (0.2326)	1.4459*** (0.2394)
*Trade*	1.4632*** (0.2760)	1.3094*** (0.2671)	1.2145*** (0.2150)	1.1470*** (0.2481)
*Locked countries*	−0.6307*** (0.1359)	−0.6258*** (0.1211)	−0.6399*** (0.1055)	−0.6441*** (0.1101)
*Unitary state*			1.2965*** (0.2711)	1.1224*** (0.3483)
*Km from Wuhan*			0.3808* (0.2266)	0.6654** (0.2786)
*Days to next election*			0.5083*** (0.1489)	0.0929 (0.1569)
Num. observations	36	45	36	45
Residual/null deviance	0.6833	0.6897	0.8316	0.7565

Notes: Standard errors in brackets.

* *p* < .1,

** *p* < .05,

*** *p* < .01.

**Figure 2:**
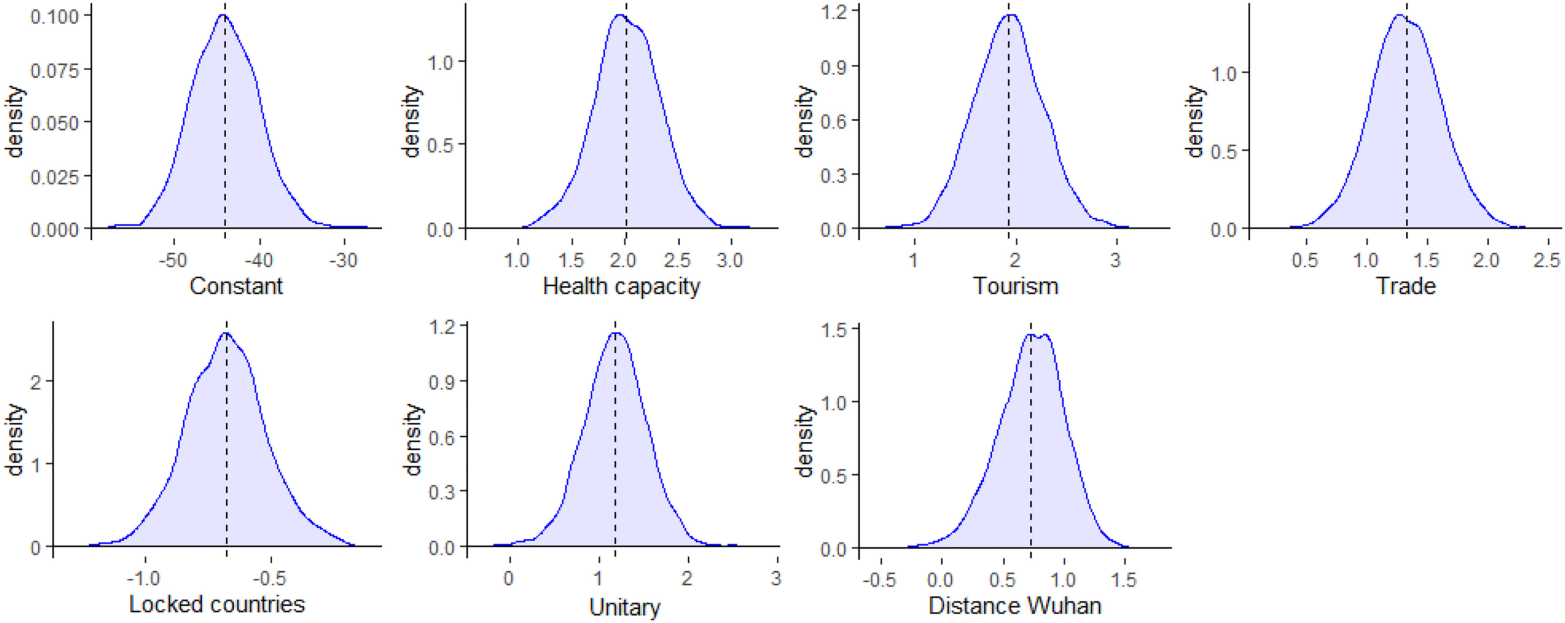
Distribution of the Parameters of the Model Using a Bayesian Estimation

Next, we carry out an additional robustness check by conducting a Bayesian estimation of the model. Low sample size can lead to less robust estimations of parameters and standard errors, thus compromising the GLM significance test, which relies on asymptotic properties of the estimators (Western and Jackman 1994). We perform the Bayesian estimation using the *brms package* available in R (Bürkner 2017) and using no prior to avoid introducing any bias. Since the *days to election* variable is not robust to the inclusion of additional countries, we include only the *Unitary* dummy and the *kilometers from Wuhan* extension. As Figure 2 shows, all parameters are robust to the Bayesian estimation.

Finally, to gain a complete understanding of the model beyond the significance of the parameters, we estimate the relative importance of each variable included in the model, using a new methodology for model interpretation suggested by Lundberg and Lee (2017, 2019)): SHAP (SHapley Additive ExPlanation) values. On synthesis, given an observation *x* = (*x*_1_, …, *x*_*J*_), the SHAP value of feature *j* on instance *x* corresponds to the way in which the concrete value of feature *j* on *x* modifies the output of the model with respect to other instances that share some features with *x* but not *j*. For a parametric model $F\left(x\right)=g\left(\sum_{j}\alpha_{j}x_{j}\right)$, where *g* is a function of the weighted features of *x*, the SHAP value corresponds to: *φ*_*j*_(*x*) = *α*_*j*_(*x*_*j*_ − *E*(*X*_*j*_)) where *X* is the set of observations and *E*(*X*_*j*_) is the average value of the *j* feature on *X*. Then, noting *N* as the total number of observations, we can estimate the relative importance of feature *j* in the model as:

$$RI_{j}=\frac{\sum_{i=1}^{n}|\varphi_{j}\left(x_{i}\right)|}{\sum_{k=1}^{J}\sum_{i=1}^{n}|\varphi_{k}\left(x_{i}\right)|}$$

Table 8 presents the relative importance of each variable in the final model, estimated using the Bayesian approach.

**Table 8:** Final Model

	Final Model (12)	Bayesian Estimate^a^	Relative Importance
Constant	−44.4938*** (3.1912)	−44.0198*** (4.0020)	
*Healthcare capacity*	2.0338*** (0.2712)	2.0172*** (0.3113)	26.6%
*Tourism*	1.9030*** (0.2568)	1.9227*** (0.3456)	20.9%
*Trade*	1.3059*** (0.2377)	1.3228*** (0.2884)	16.1%
*Locked countries*	−.6670*** (0.1216)	−0.6773*** (0.1606)	19.5%
*Unitary state*	1.2142*** (0.3004)	1.1824*** (0.3554)	11.0%
*Km from Wuhan*	0.7701*** (0.2308)	0.7288*** (0.2737)	5.9%
Num. observations	36	36	
Residual/null deviance	0.7923	0.7945	

Notes: Standard errors in brackets.

* *p* < .1,

** *p* < .05,

*** *p* < .01.

a The Reset test for functional form or omitted variables with a polynomial fitting of degree 4 does not reject the null hypothesis (*p* value .6049). Therefore, the functional form is correct, and the estimates do not suffer from omitted variables.

## Discussion and Policy Implications

All governments have been overwhelmed by the pandemic and forced to implement hard measures to avoid a complete healthcare‐system collapse and its associated fatalities, which would have led to an even more negative valuation of their policy responses. According to our theoretical model, once a government has a clear expectation that it will have to implement hard measures, the choice to enact them immediately strictly dominates the choice to delay them. For this reason, the fact that healthcare‐system capacity and cost‐related variables have a significant influence on reaction time has a very relevant implication: they negatively affected government strategy.

Because initial expectations did not match reality (otherwise, governments would have had not taken hard measures), governments with strong healthcare systems were overconfident about their ability to fight the outbreak and did not immediately implement hard measures. The associated economic costs created a fear of excessive economic damage. Both overconfidence and economic fears delayed the implementation of hard measures, increasing overall costs. Notice that implementing “hard measures” as a result of a “rational” (cost–benefit based) decision process with incomplete information might not have been “optimal” in all countries. Whether hard measures had been or not optimal in each case would need an ex‐post evaluation of effects, which is beyond the scope of our study, and estimating the actual impact of the pandemic on the fatality rates and economic costs associated with agile and slow policy responses is a question for future research, as complete data will not be available until the COVID‐19 crisis is over.

Our results are empirically robust and supported by frequent public statements made by political leaders. Indeed, overconfidence in healthcare‐system capacity has been described as one of the main causes of policy‐response delays by global healthcare experts, including Pedro Alonso, Director of the World Health Organization's Malaria Program, who said on May 6 that Western pride prevented most advanced countries from reacting quickly.

As the pandemic triggered a decision‐making process based on incomplete information, variables related to additional information (e.g., the policy responses of other countries) and valuation of risk (proximity bias) are key factors, directly accounting for 25 percent of the total.

Finally, multilevel governance is also relevant. Federal countries, which are more decentralized and better at fostering political collaboration, were more agile than unitary states.

We are aware that our identification strategy cannot draw strong claims of causal relations from these empirical results; this is a limitation of the present research. However, we believe that our theoretical model (built on a very simple hypothesis), when combined with many statements and observations that support a causal relationship—made by policy leaders and healthcare experts—can reduce this limitation.

There is a wide consensus that strong healthcare‐system capacity improves social welfare, while high levels of trade and tourism are important engines of economic growth. However, these benefits risk biasing governments, particularly in the context of crisis management under incomplete information. Ballesteros and Kunreuther (2018, p. 9), in their analysis of organizational decision‐making in the face of uncertainty shocks, warn that “the riskification of uncertainty leads to the delusion that increasing formal insurance take‐up is a sufficient mechanism to reduce vulnerability against uncertainty shocks.” An important policy implication emerges from this analysis. The COVID‐19 pandemic has generated frequent demands to increase health expenditure. Indeed, such expenditure may improve health‐system performance on a regular day‐to‐day basis, as long as the additional capacity meets positive social cost–benefit requirements. However, it will not provide full insurance for managing future pandemics, as strong healthcare‐system capacity can induce governments to make riskier decisions, particularly under incomplete information.

## Conclusion

In this study, we have built a theoretical model and used it to design and implement an empirical strategy, analyzing why some countries took longer than others to implement lockdown measures. In other words, we set out to discover the drivers of policy‐response agility during the COVID‐19 outbreak.

Our findings show that welfare variables, involving a cost–benefit analysis of policy responses, were the most significant drivers. Together, healthcare capacity and expected economic costs accounted for around 65 percent of the total importance. If governments have had complete information, we would have expected these factors not to be relevant; once governments know for certain that they must implement hard measures, they clearly prefer to anticipate rather than to delay. The importance of these variables therefore indicates that governments may have been biased in their risk assessment of the pandemic by healthcare‐system capacity and the fear of direct economic costs.

In addition, information about the progress of the pandemic was a key driver, accounting for around 25 percent of total relevance. The more information governments had access to, the more agile they were in their policy responses. Last but not least, we found empirical evidence that decision‐making processes and individual actors were also relevant. Decentralized federal states, which promote political competition, were more agile than unitary states.

While we found no evidence that concerns related to inequality, poverty, or trust in government shaped policy‐response agility, they may have influenced the severity of instituted measures. Hence, these topics deserve future research. Future studies should analyze in depth the wide range of policy responses in the United States, given its complex governance, institutional design, and comparatively high level of political and ideological polarization.

## Notes

1 Simulations done using the R *EpiModel* package for SIR models, with a population of size N = 1,000, 1 initial case, and an initial reproductive number of 1.85 (assuming 6.1 days of effective transmission, before the individual is quarantined, hospitalized, recovered or died, and an effective contact rate of 0.3), lead to achieving the pick of the pandemic after around 35 days, with daily new cases reaching the 3% of the total population.

2 We are aware that choosing a national scale may mask sub‐national governments’ activity, and this is a relevant issue in federal countries, as discussed in Downey and Myers (2020) when comparing the US and Australia. Unfortunately, most data we use in our empirical analysis (e.g. incidence rate) are only available at the national level, and this is a constraint to consider also subnational levels in our empirical exercise. This might be particularly relevant for the case of the US, where debate on executive federalism (Bulman‐Pozen 2016; Eleazar 1993) has emphasized that the absence of a formal coordinating institution has impact on sub‐national policy adoption in the US (Downey and Myers 2020). Because of this, on the one hand, we have included a categorical variable on whether the response was Unitarian or Federal (see the subsection “Type of decision‐maker”). On the other hand, we re‐run our estimations in Table 3 also excluding the US, and the results remained the same.

## 1

**Table A1:** List of Date of First Hard Measures

Country	Date Hard Measures	Description
Australia	3/19/2020	Border closure; closure of some nonessential shops; 4 Square meter rule
Austria	3/15/2020	Nationwide lockdown (including closure of schools), closure of all nonessential shops, ban of public gatherings
Belgium	3/12/2020	Closure of schools (but not universities), discos, cafes and restaurants, and the cancelation of all public gatherings for sporting, cultural or festive purposes
Bulgaria	3/13/2020	Closure of nonessential shops and workplaces, mandatory quarantine for all people coming from most affected countries
Canada	3/16/2020	Border closure, states of emergency including closure of nonessential shops, ban of public gathering, etc. in all Canadian states but Manitoba, New Brunswick and Nova Scotia
Chile	3/16/2020	Border closure, state of emergency, partial lockdowns in affected cities and regions, closure of schools with at least one case.
Croatia	3/17/2020	Closure of most nonessential shops, schools, and universities; 14‐days mandatory quarantine for people coming from affected countries, border closure
Cyprus	3/13/2020	Border closure, ban of public gatherings
Czech Rep.	3/12/2020	Border closure, nationwide curfew, schools suspended, closure of nonessential shops
Denmark	3/11/2020	Closure of schools and universities, banning of public gatherings, home‐work public sector, border closure
Estonia	3/13/2020	Border closure, closure of schools, ban of public gatherings, closure of recreation and leisure shops
Finland	3/16/2020	Closure of schools and universities, banning of public gatherings, shut‐down of most government‐run facilities (libraries, etc.)
France	3/16/2020	Closure of most nonessential shops, ban of public gatherings, closure of schools and institutes of higher education
Germany	3/16/2020	Closure of education institutions, ban of public gatherings, closure of nonessential shops in some states
Greece	3/13/2020	Closure of education institutions, ban of public gatherings, closure of cafes, bars, museums, shopping centers, sports facilities and restaurants, border closure with limiting countries and affected countries
Hungary	3/15/2020	Closure of education institutions, bars, restaurants, cafes, public events, border closure
Iceland	3/13/2020	Closure of educational institutions, ban of public gatherings and events
Ireland	3/24/2020	Closure of education institutions, bars, and public houses
Israel	3/14/2020	Closure of education institutions, most nonessential retail, ban of public gatherings
Italy	3/8/2020	Complete lockdown north Italy, ban public gatherings
Japan	3/5/2020	Closure of education institutions and extension of the law's emergency measures for an influenza outbreak to include COVID‐19
Korea	2/20/2020	Border closure with China, massive testing and surveillance, partial lockdowns on more affected areas
Latvia	3/14/2020	Closure of educational institutions, ban of public events
Lithuania	3/12/2020	Closure of educational institutions, ban public gatherings, borders closure, closure of nonessential shops
Luxembourg	3/15/2020	Closure of nonessential shops, ban of public gatherings, closure educational institutions
Mexico	3/26/2020	Closure of nonessential shops and nonessential activities, ban of public gatherings, closure of educational institutions
Netherlands	3/15/2020	Closure of educational institutions; closure of cafés, restaurants, sports clubs, saunas, sex clubs, coffee shops, museums; ban of public events
New Zealand	3/23/2020	Border closure, ban of public gatherings, closure of all venues and enforcement of telework whenever possible
Norway	3/12/2020	Closure of kindergartens, schools, universities, and some none‐essential shops (bars, restaurants, pubs, clubs, among others)
Poland	3/11/2020	Closure of all schools and universities, gathering restrictions and closure of cultural institutions, such as philharmonic orchestras, operas, theaters, museums, and cinemas
Portugal	3/12/2020	State of emergency; closure of establishments in the hospitality sectors such as restaurants, pubs, bars; public gathering restrictions; closure of all education institutions (from kindergartens to universities)
Romania	3/9/2020	Border closure with affected regions; all schools, kindergartens, and universities closed
Slovak Rep.	3/15/2020	Implementation of state of emergency with all nonessential stores closed, closure of all schools and 14 days quarantine for people arriving from Slovakia from Italy, China, South Korea
Slovenia	3/15/2020	Closure of all educational institutions, bars and restaurants, and gathering restriction
Spain	3/14/2020	State of emergency declared, with closure of all educational institutions, hospitality sector establishments. People are to remain locked down in their homes except for essential activities
Sweden	3/27/2020	Reunion right restriction to 50 people
Switzerland	3/13/2020	Closure of all educational institutions and gathering restriction of more than 100 people, cancelation of all sport events
Turkey	3/12/2020	Closure of all schools and universities, travel bans, and border closure with affected countries
U. Kingdom	3/18/2020	Closure of all schools, restaurants, pubs/clubs, and indoor leisure facilities
United States	3/15/2020	State of emergency >25 states with closure of education institutions, curfew population, borders closure (main affected areas, including EU)
Serbia	3/15/2020	Closure of all education institutions from kindergartens to universities, ban public gathering, border closure
N. Macedonia	3/11/2020	Closure of all education institutions from kindergartens to universities, border closure, and ban of public gatherings
Albania	3/8/2020	Closure of education institutions, gyms, bars, and restaurants
Malta	3/12/2020	Closure of all schools, university and childcare, bars, restaurants and gym, mandatory quarantine to travelers from any country
Montenegro	3/13/2020	Closure education institution, bars and borders; ban on public gatherings

Notes: Sweden never applied a lockdown strategy, preferring to follow a recommendation‐based approach. The moment of policy response was March 27th, when the government banned public gatherings of more than 50 people and imposed up to 6‐month prison sentences on those who broke the ban. This was the hardest measure approved in Sweden, 10 days after all European countries had closed their borders. At that point, two restrictions were in place: Sweden was isolated by its neighbors and public gatherings were prohibited.

**Table A2:** Correlation Matrix

	Incidence Rate	Healthcare Capacity	Tourism	Trade	Locked Countries	Unitary	Km Wuhan
Incidence rate at policy response	—						
Healthcare capacity (ppp)	45%	—					
Tourism	31%	−15%	—				
Trade	18%	−8%	−32%	—			
Locked Countries	−19%	−57%	9%	12%	—		
Unitary	11%	−32%	−3%	19%	25%	—	
KmWuhan	17%	4%	7%	16%	7%	−27%	—

Notes: We include Km from Wuhan and Unitary because they are used in the final model. The average variance inflation factor of the covariates is 1.34 and the highest is 1.62.

**Table A3:** Estimated Parameters of the Base Model with Second‐Order Costs

	Base Model (1)	(A1)	(A2)	(A3)
Constant	−35.7629*** (3.2399)	−40.5855*** (4.1372)	−36.5993*** (3.2741)	−35.7945*** (3.2279)
*Healthcare capacity*	1.8814*** (0.3199)	2.0900*** (0.3419)	1.9468*** (0.3226)	1.8941*** (0.3201)
*Tourism*	1.7654*** (.3086)	2.4624*** (0.2932)	1.6669*** (0.3129)	1.9072*** (0.3062)
*Trade*	1.4632*** (0.2760)	2.0031*** (0.2700)	1.4973*** (0.2718)	1.5725*** (0.2746)
*Locked countries*	−0.6307*** (0.1359)	−0.6779*** (0.1441)	−0.6067*** (0.1296)	−0.7077*** (0.1417)
*% contribution MSMEs*		0.0143 (0.0173)		
*% unemployment*			0.0544 (0.0400)	0
*% population > 65*				−0.0454 (0.03726)
				
N. Observations	36	33	36	36
Residual/null deviance	0.6833	0.7369	0.6950	0.6929

Notes: Standard errors in brackets.

* *p* < .1,

** *p* < .05,

*** *p* < .01.

**Table A4:** Estimated Parameters of Alternative Specifications for Ideology and Trust

	(13)	(A4)	(A5)	(A6)
Constant	−44.4938*** (3.1912)	−44.6347*** (4.2258)	−47.2778*** (4.1158)	−44.3957*** (3.6499)
*Healthcare capacity*	2.0338*** (0.2712)	2.0284*** (0.3075)	2.2444*** (0.3301)	1.9620*** (0.2577)
*Tourism*	1.9030*** (0.2568)	1.9105*** (0.2638)	1.9475*** (0.2577)	1.9121*** (0.2577)
*Trade*	1.3059*** (0.2377)	1.3131*** (0.2801)	1.3302*** (0.2427)	1.3184*** (0.2392)
*Locked countries*	−0.6670*** (0.1216)	−0.6723*** (0.1218)	−0.6108*** (0.1279)	−0.6794*** (0.1256)
*Unitary state*	1.2142*** (0.3004)	1.2175*** (0.3106)	1.3274*** (0.3276)	1.2263*** (0.3015)
*Km from Wuhan*	0.7701*** (0.2308)	0.7851*** (0.2317)	0.8057*** (0.2296)	0.8040*** (0.2438)
*GINI index*		−0.0073 (2.6575)		
*% poverty*			0.0216 (0.0243)	
*Trust*				0.0289 (0.1443)
N. Observations	36	36	36	36
Residual/null deviance	0.7923	0.7924	0.7926	0.7911

Notes: Standard errors in brackets.

* *p* < .1,

** *p* < .05,

*** *p* < .01.
